# A Survey of Generational Trends: Practitioner Insights Into Aesthetic Preferences

**DOI:** 10.1111/jocd.70397

**Published:** 2025-08-12

**Authors:** Thibault Trévidic, Patrick Trévidic, Nabila Azib, Gisella Criollo, Shady Ismail, Amin Sharobim, Benji Dhillon

**Affiliations:** ^1^ Expert2expert Group Budapest Hungary; ^2^ Expert2expert Group Paris France; ^3^ Expert2expert Group Rabat Morocco; ^4^ Expert2expert Group Cairo Egypt; ^5^ Define Clinic London UK

**Keywords:** aesthetic medicine, beauty, centennials, gen Z, millennials, preferences, survey

To the Editor,

We conducted an observational survey among aesthetic practitioners, highlighting their differing attitudes and expectations. Generation Y (Millennials, born 1981–1996) and Generation Z (Centennials, born 1997–2012) mark a sociological break in their attitude to aesthetic medicine from previous generations. The growing influence of social media has revolutionized their approach to aesthetics, with a greater emphasis on earlier maintenance and prevention [1, 2]. The objective of the survey reported here was to assess the opinions and experiences of aesthetic practitioners of these two generations, to guide our approach when treating these groups.

## 1 Materials and Methods

A cross‐sectional observational survey assessing the experience of aesthetic healthcare professionals (HCPs) across the two generations was performed in 2023. HCPs were recruited to complete the survey through the Expert2expert Faculty Network. Participating HCPs completed a 23‐question interview on their experience, patient preferences, and general perception statements relating to patient recruitment, satisfaction, retention, and finances (Supporting Information S1).

## 2 Results

In total, 40 HCPs were interviewed, and full results are shown in Supporting Information S1. The majority were > 35 years old (97.5%), with > 10 years' experience (70%) and undertaking > 50 injections per month (75%). On average, a median proportion of “31%–40%” (11%–80%) of their database patients were Millennials and “11%–20%” (1%–40%) Centennials.

**FIGURE 1:**
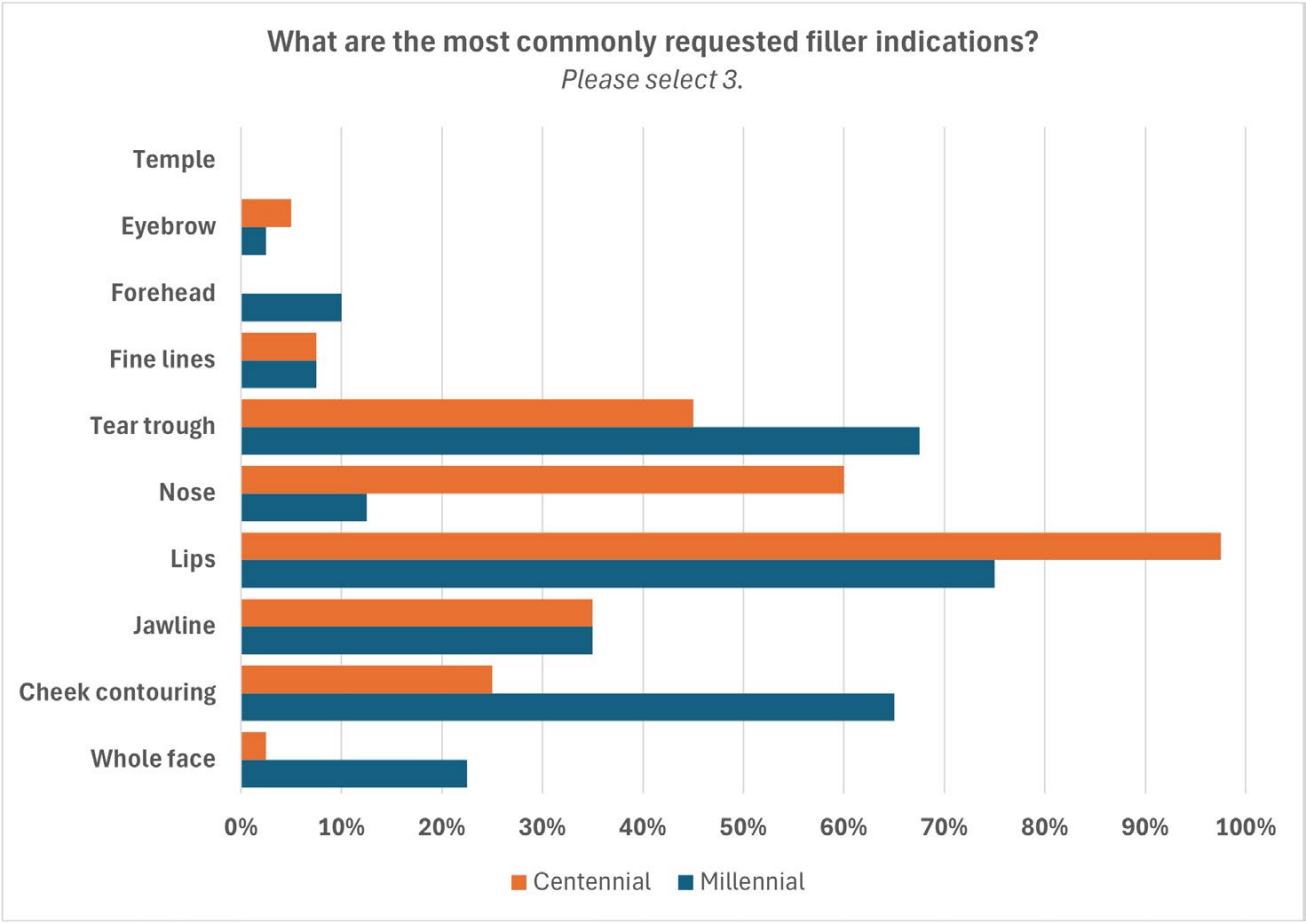
Most requested filler indications for Millennials (blue) and Centennials (orange).

When asked to rank the most requested treatments, Millennials requested botulinum toxin, fillers, and skincare, while Centennials requested fillers, skincare, and botulinum toxin. The most requested filler indications are shown in Figure 1. The average volume used per treatment plan (over 4–6 weeks) was higher in the Millennial group, with 2–3 syringes compared to the more commonly used one syringe to complete treatment, and the products used were in line with indications.

The results of the questions relating to patient recruitment, satisfaction, retention, and finances are shown in Table 1. Most practitioners did not find it technically difficult to treat or achieve patient satisfaction with either generation; although a significant minority (40%) found Centennials more difficult to satisfy (Table 1).

**TABLE 1:** Patient attitudes.

	Millennials	Centennials
How did the patients find you?		
Social media	27.5%	60.0%
Relatives	12.5%	20.0%
Friends	60.0%	20.0%
Do you think patients from this generation are more difficult to treat than others?		
Yes	12.5%	35.0%
No	87.5%	65.0%
Do you think patients from this generation are more difficult to satisfy than others?		
Yes	27.5%	40.0%
No	72.5%	60.0%
Do you think patients from this generation are more difficult to retain than others?		
Yes	27.5%	77.5%
No	72.5%	22.5%
Do you think patients from this generation are more limited in terms of budget than others?		
Yes	22.5%	80.0%
No	77.5%	20.0%

## 3 Discussion

The objective of this survey was to identify differences in treatment patterns and expectations across generations. Our findings revealed that Millennials primarily sought treatments for wrinkle reduction, in line with other studies showing a rise in botulinum toxin injections with age [3]. In contrast, the most requested treatment among Centennials was dermal fillers, usually to address a perceived genetic or structural issue, mirroring data showing that they most frequently request nose procedures, in line with the Alpha generation (born 2010–present) [2, 3].

These results support published data suggesting that younger generations are more focused on changing or enhancing their appearance, while older generations seek rejuvenation treatments [3]. Although Millennials generally “consumed” more products than Centennials in this setting, likely due to greater financial stability and treatment needs, this was less pronounced than expected and proportional to the areas treated. Given that Centennials may have more limited budgets for cosmetic procedures, it is important that practitioners carefully tailor their plans to ensure that treatments are efficient, results‐driven, and visibly effective; ideally achieved with fewer sessions.

Interestingly, although social media channels were an important promotion source, there was cross‐generation variation (27.5% vs. 60%), reflecting differing social network usage. While social media is a dominant recruitment method for Centennials, information can be misleading. Practitioners should take care to have open conversations about realistic expectations and provide evidence‐based guidance to distinguish between realistic outcomes and unattainable goals driven by online trends.

Clinically, most HCPs surveyed did not find it technically difficult to treat either generation, suggesting that techniques are adaptable as patients age. Despite younger generations potentially being influenced by unrealistic images on social media, most survey participants felt confident in their ability to achieve patient satisfaction, suggesting that they are well‐trained to effectively manage these patients.

Notably, Millennials exhibited greater treatment loyalty, with 77.5% of practitioners reporting difficulty retaining Centennial patients. This generational difference may be attributed to low brand loyalty, budget constraints, social media influence, or different expectations around continuity of care. While specific causes were not part of this survey, this appears to be a broader generational trend, not limited to injectable treatments. To mitigate this form of “medical tourism” or care fragmentation, in our experience, practitioners should build trust and long‐term engagement by providing clear practical explanations of treatments, guiding rather than challenging patient preferences, and showing them examples of achievable outcomes.

Key limitations of this survey include potential respondent and generational biases. Given that most survey respondents were professionals over the age of 35, perceptions of younger patients' behavior and satisfaction may be skewed. The small sample size, composed primarily of Dermatologists and Plastic Surgeons recruited via the Expert2Expert and Faculty Network platforms, may also limit the generalizability of the findings. Additionally, the use of descriptive statistics and retrospective self‐reported data introduces potential recall bias, limiting the interpretation of the results.

## 4 Conclusions

This survey highlights the different attitudes of these two generations to allow us to better understand and treat them effectively as they become increasing consumers of aesthetic medicine.

## Author Contributions

All authors contributed equally to this article, including the concept, methodology, formal analysis, and the writing, reviewing, and editing process.

## Conflicts of Interest

The authors declare no conflicts of interest.

## Supporting information


**Data S1:** jocd70397‐sup‐0001‐Supinfo1.pdf.

## Data Availability

The data that support the findings of this study are available from the corresponding author upon reasonable request.
