# Supplementary material for: A Survey of Generational Trends: Practitioner Insights Into Aesthetic Preferences
Source: J Cosmet Dermatol. 2025 Aug 12;24(8):e70397. doi: 10.1111/jocd.70397 (PMC12340661; doi:10.1111/jocd.70397)
Supplement: Supplementary file 1 — Data S1: jocd70397‐sup‐0001‐Supinfo1.pdf. [file JOCD-24-e70397-s001.pdf]

Millennials and Centennials Survey

Based on your own experience, please could you answer these questions?
We received 40 answers.

1. In your patient database, what percentage of your patients are Millennials (27–42 years old)?

1–10% = 0 (0%)
11–20% = 4 (10%)
21–30% = 10 (25%)
31–40% = 6 (15%)
41–50% = 5 (12.5%)
51–60% = 7 (17.5%)
61–70% = 6 (15%)
71–80% = 2 (5%)
81–90% = 0 (0%)
91–100% = 0 (0%)

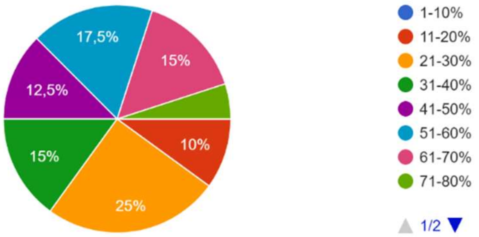

2. In your patient database, what percentage of your patients are Centennials (18–26 years old)?

1–10% = 17 (42.5%)
11–20% = 13 (32.5%)
21–30% = 9 (22.5%)
31–40% = 1 (2.5%)
41–50% = 0 (0%)
51–60% = 0 (0%)
61–70% = 0 (0%)
71–80% = 0 (0%)
81–90% = 0 (0%)
91–100% = 0 (0%)

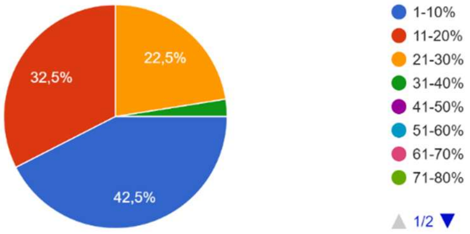

3. Which of the following treatments is the most commonly desired for Millennials? Please rank from 1 to 3.

1° Fillers — 2° Botulinum toxin — 3° Skincare = 8 (20%)

1° Fillers — 2° Skincare — 3° Botulinum toxin = 4 (10%)

1° Botulinum toxin — 2° Fillers — 3° Skincare = 19 (47.5%)

1° Botulinum toxin — 2° Skincare — 3° Fillers = 5 (12.5%)

1° Skincare — 2° Fillers — 3° Botulinum toxin = 2 (5%)

1° Skincare — 2° Botulinum toxin — 3° Fillers = 2 (5%)

4. Which of the following treatments is the most commonly desired for Centennials? Please rank from 1 to 3.

1° Fillers — 2° Botulinum toxin — 3° Skincare = 9 (22.5%)

1° Fillers — 2° Skincare — 3° Botulinum toxin = 13 (32.5%)

1° Botulinum toxin — 2° Fillers — 3° Skincare = 4 (10%)

1° Botulinum toxin — 2° Skincare — 3° Fillers = 5 (12.5%)

1° Skincare — 2° Fillers — 3° Botulinum toxin = 3 (7.5%)

1° Skincare — 2° Botulinum toxin — 3° Fillers = 6 (15%)

5. What are the most common requested filler indications for Millennials? Please select 3.

Whole face = 9 (22.5%)
Cheek contouring = 26 (65%)
Jawline = 14 (35%)
Lips = 30 (75%)
Nose = 5 (12.5%)
Tear trough = 27 (67.5%)
Fine lines = 3 (7.5%)
Forehead = 4 (10%)
Eyebrow = 1 (2.5%)
Temple = 0 (0%)

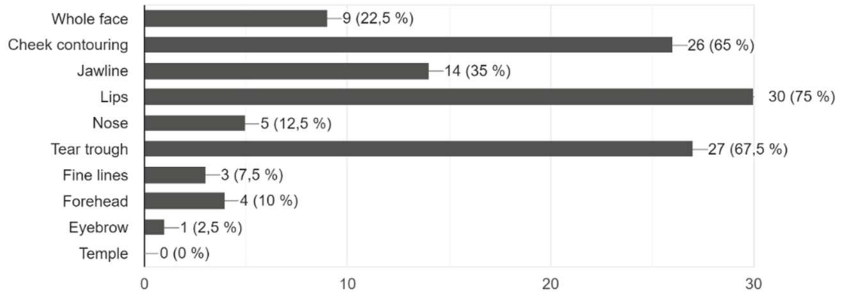

6. What are the most common requested filler indications for Centennials? Please select 3.

Whole face = 1 (2.5%)
Cheek contouring = 10 (25%)
Jawline = 14 (35%)
Lips = 39 (97.5%)
Nose = 24 (60%)
Tear trough = 18 (45%)
Fine lines = 3 (7.5%)
Forehead = 0 (0%)
Eyebrow = 2 (5%)
Temple = 0 (0%)

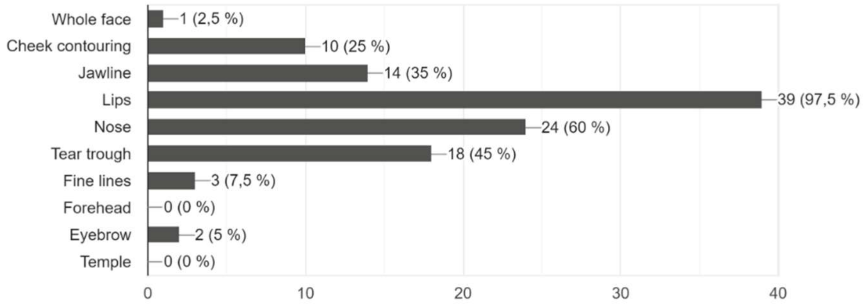

7. What is the average volume you use for Millennials in one treatment plan (may be over 4–6 weeks)?

1 syringe = 7 (17.5%)
2–3 syringes = 20 (50%)
3–4 syringes = 8 (20%)
4 syringes or more = 5 (12.5%)

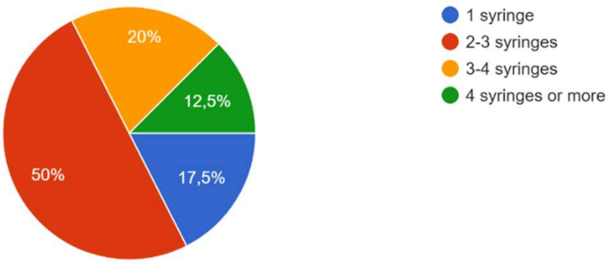

8. What is the average volume you use for Centennials in one treatment plan (may be over 4–6 weeks)?

1 syringe = 24 (60%)
2–3 syringes = 16 (40%)
3–4 syringes = 0 (0%)
4 syringes or more = 0 (0%)

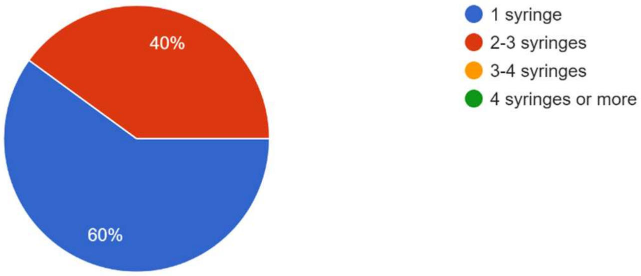

9. **What is the most common product choice in Millennials?**

Redensity 1 = 0 (0%)
Redensity 2 = 3 (7.5%)
Ultradeep = 5 (12.5%)
Kiss = 4 (10%)
RHA 1 = 1 (2.5%)
RHA 2 = 10 (25%)
RHA 3 = 5 (12.5%)
RHA 4 = 12 (30%)

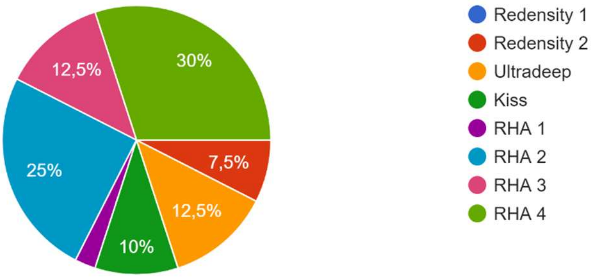

10. **What is the most common product choice in Centennials?**

Redensity 1 = 0 (%)
Redensity 2 = 3 (7.5%)
Ultradeep = 1 (2.5%)
Kiss = 8 (20%)
RHA 1 = 1 (2.5%)
RHA 2 = 16 (40%)
RHA 3 = 10 (25%)
RHA 4 = 1 (2.5%)

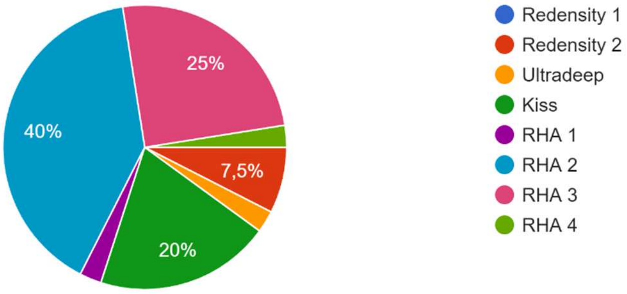

11. **How did Millennial patients know you?**

Social media = 11 (27.5%) Relatives = 5 (12.5%) Friends = 24 (60%)

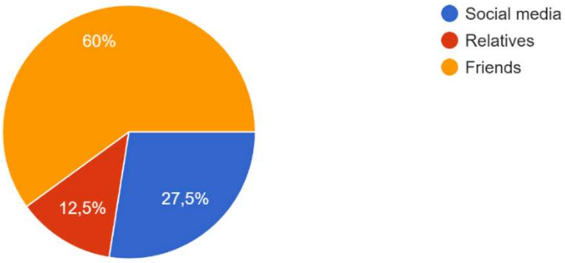

12. **How did Centennial patients know you?**

Social media = 24 (60%) Relatives = 8 (20%) Friends = 8 (20%)

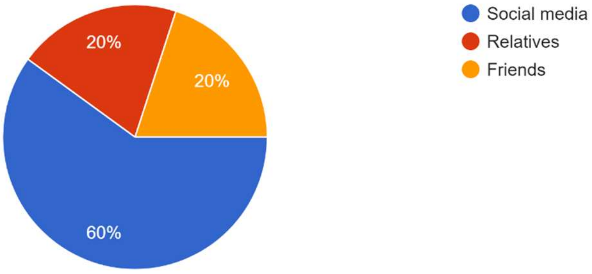

13. **Do you think Millennial patients are more difficult to treat than others?**

Yes = 5 (12.5%) No = 35 (87.5%)

14. **Do you think Centennial patients are more difficult to treat than others?**

Yes = 14 (35%) No = 26 (65%)

15. Do you think Millennial patients are more difficult to satisfy than others?

Yes = 11 (27.5%) No = 29 (72.5%)

16. Do you think Centennial patients are more difficult to satisfy than others?

Yes = 16 (40%) No = 24 (60%)

17. Do you think Millennial patients are more difficult to retain than others?

Yes = 11 (27.5%) No = 29 (72.5%)

18. Do you think Centennial patients are more difficult to retain than others?

Yes = 31 (77.5%) No = 9 (22.5%)

19. Do you think Millennial patients are more limited in terms of budget than others?

Yes = 9 (22.5%) No = 31 (77.5%)

20. Do you think Centennial patients are more limited in terms of budget than others?

Yes = 32 (80%) No = 8 (20%)

21. How much experience do you have with injections?

<5 years = 4 (10%)
6–10 years = 8 (20%)
11–15 years = 13 (32.5%)
16–20 years = 6 (15%)
>21 years = 9 (22.5%)

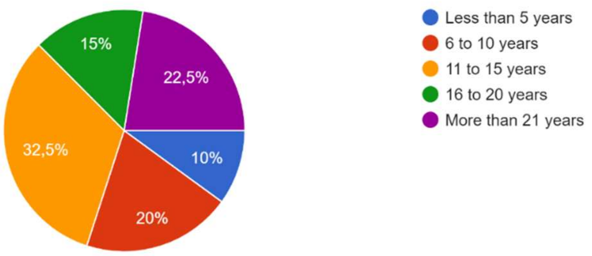

22. How many injections are you doing each month?

<10 = 1 (2.5%)
11–30 = 4 (10%)
31–50 = 5 (12.5%)
51–80 = 6 (15%)
81–100 = 7 (17.5%)
>100 = 17 (42.5%)

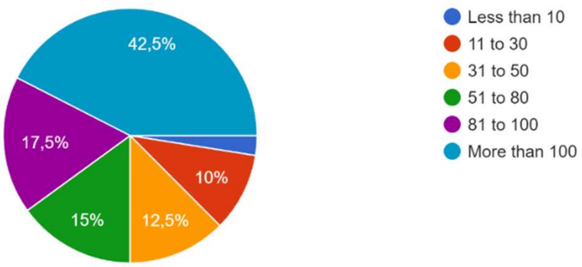

23. How old are you?

<34 years = 1 (2.5%)
35–44 years = 18 (45%)
45–54 years = 12 (30%)
55–64 years = 7 (17.5%)
>65 years = 2 (5%)

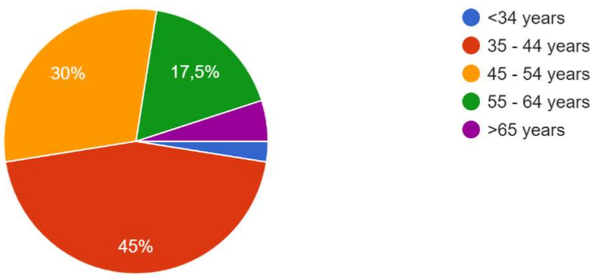

Thank you for answering this survey!
